# The ability of anexelekto (AXL) expression and *TERT* promoter mutation to predict radioiodine-refractory differentiated thyroid carcinoma

**DOI:** 10.1186/s13000-025-01643-0

**Published:** 2025-04-16

**Authors:** Hasrayati Agustina, Tutik Nur Ayni, Yohana Azhar, Erwin Affandi Soeriadi, Bethy Suryawathy Hernowo

**Affiliations:** 1 Department of Anatomic Pathology, Dr. Hasan Sadikin Hospital, Bandung, Indonesia; 2 Department of Surgery Subdivision Oncology Surgery, Dr. Hasan Sadikin Hospital, Bandung, Indonesia; 3 Department of Nuclear Medicine and Molecular Theranostic, Dr. Hasan Sadikin Hospital, Bandung, Indonesia; 4 Faculty of Medicine, Universitas Padjadjaran, Bandung, Indonesia

**Keywords:** Anexelekto, *TERT* promoter, Radioiodine refractory, Differentiated thyroid carcinoma

## Abstract

**Background:**

Differentiated thyroid carcinoma (DTC) generally has a favourable prognosis with standard treatments; however, the risks of local recurrence and distant metastases remain a concern, affecting a substantial proportion of patients. Radioactive iodine (RAI) refractoriness further complicates DTC management, leading to substantially reduced survival rates. In this study, we aimed to identify anexelekto (AXL) expression and *TERT* promoter mutation as potential predictors of RAI-refractory DTC.

**Methods:**

We conducted a retrospective analysis of 81 DTC patients who underwent thyroidectomy and received at least two courses of RAI therapy. After a median follow-up period of 30 months (range: 6–60 months), therapy response was categorized as nonrefractory or refractory. AXL expression and *TERT* promoter mutation were evaluated in all patients to discern any associations with the development of RAI refractoriness.

**Results:**

The overall prevalence of refractory RAI in DTC patients was 44.4% (36/81). AXL expression was high in 30/36 patients (83.3%) with RAI-refractory DTC and negative/low in 24/45 patients (53.3%) with non-RAI-refractory DTC (OR adjusted: 44.98, CI 95%: 1.41-1439.03, *p* = 0.031). *TERT* promoter mutation occurred in 21/36 (58.3%) RAI-refractory DTCs and in 2/45 (4.4%) non-RAI-refractory DTCs (OR adjusted: 10.95, CI 95%: 1.06-112.92, *p* = 0.044). Despite similar age, sex, and histological type distributions between the RAI-refractory and non-RAI-refractory groups, significant differences in clinicopathological characteristics emerged. Multivariate analysis confirmed that aggressive subtype, elevated AXL expression, and *TERT* promoter mutation independently correlated with RAI-refractory status.

**Conclusions:**

Our predictive model highlights the association of elevated AXL expression, *TERT* promoter mutation, and an aggressive tumour subtype with the risk of RAI refractoriness. This information has the potential to aid in making informed treatment decisions. Furthermore, AXL is a potential therapeutic target for RAI-refractory disease.

## Background

Differentiated thyroid carcinoma (DTC) is derived from abnormal follicular cells and constitutes the majority of all thyroid cancer cases, accounting for approximately 90–95% [1]. This category encompasses papillary thyroid carcinoma (PTC), follicular thyroid carcinoma (FTC), Hurthle cell/oncocytic carcinoma (OCA), and poorly differentiated thyroid carcinoma (PDTC) [2]. The initial treatment approach for DTC involves thyroidectomy and, when necessary, central or lateral compartment neck dissection. Additionally, radioactive iodine (RAI) is administered to patients who present intermediate- to high-risk features along with other relevant clinical factors [3]. While the majority of DTC patients respond favourably to standard therapeutic modalities, including surgery, selective RAI treatment, and thyroid-stimulating hormone (TSH) suppression therapy, there is a considerable risk of local recurrence (up to 30%) and distant metastases (up to 10%). Alarmingly, among these patients, approximately two-thirds exhibit an early or gradual loss of iodine uptake, indicative of a dedifferentiated state termed RAI-refractory. This clinical scenario raises major concerns, given the exceedingly low 10-year survival rate (less than 10%) and a median overall survival of merely 2.5–3.5 years [1, 4]. The efficacy of RAI therapy is significantly compromised for patients who do not respond or who become refractory to ^131^I [5]. Hence, early identification of RAI-refractory DTC is paramount to avoid unnecessary RAI therapy and to explore potentially more effective treatments, such as tyrosine kinase inhibitors.

Several prior studies have focused on identifying clinicopathological characteristics and mutational status as predictors of RAI-refractory DTC; however, few have investigated these findings in the context of targeted therapy. Several studies have reported high expression levels of anexelekto (AXL), a receptor tyrosine kinase associated with cell proliferation and survival in PTC patients [6–8]. Importantly, AXL expression was positively correlated with the incidence of RAI-refractory patients and the persistence or recurrence of the disease. AXL is not only regarded as a promising prognostic biomarker in malignancies but also as a potential target for anticancer therapies [6, 9–11].

Concurrently, molecular analysis has provided valuable insights into the prediction of RAI-refractory DTC. Notably, both the *BRAF*^*V600E*^ mutation and *TERT* promoter mutation have emerged as significant predictors [12]. Additionally, Yang et al. [13] demonstrated the association between *TERT* promoter mutation and RAI-refractory distant metastatic DTC. Compared with the *BRAF*^*V600E*^ mutation, the *TERT* promoter mutation had a more pronounced negative influence on radioiodine uptake. Among the most prevalent genetic alterations in the TERT promoter region are two hotspot mutations, namely, c.-124 C > T (C228T) and c.‐146 C > T (C250T). These mutations generate binding sites for E‐twenty‐six (ETS) transcription factors, resulting in increased *TERT* transcription [14]. The canonical and noncanonical activities of *TERT* confer upon cancer cells the ability to overcome limitations in cell division, endowing them with immortality and heightened resistance to various drugs [15]. In light of these findings, the present study comprehensively analysed clinicopathological characteristics, AXL expression, and *TERT* promoter mutation as predictive factors for RAI-refractory DTC.

## Methods

### Patients and clinicopathological data

This retrospective analysis involved 81 patients with differentiated thyroid carcinoma (DTC) who underwent total or near-total thyroidectomy at Hasan Sadikin General Hospital, Bandung, Indonesia, between January 2016 and December 2021. The procedures included thyroidectomy with or without central or lateral compartment neck dissection. Patients were referred for at least two courses of radioactive iodine (RAI) therapy after the initial treatment.

Radioactive iodine (RAI) therapy is adjusted to the patient’s risk of recurrence according to the 2015 American Thyroid Association (ATA) criteria. The dose given is between 100 and 150 mCi per therapy, with 6-month intervals. To optimise isotope uptake, RAI should be given after serum thyroid-stimulating hormone (TSH) level reach ≥ 30 µIU/ml. Post therapy whole-body scans were conducted after 72–96 h of RAI administration. The case criteria for RAI-refractory patients were based on the 2015 ATA management guidelines, considering various classifications: malignant/metastatic tissue never containing RAI, loss of RAI concentration after previous evidence of RAI-avid disease, RAI concentration in some lesions but not in others, or metastatic disease progression despite substantial RAI concentration. Disease progression was defined as per the Response Evaluation Criteria in Solid Tumours (RECIST) v1.1 [16]. Despite RAI uptake, some patients were also classified as RAI refractory, especially if the treatment did not prove effective even after use of multiple additional radioactive iodine agents (600 mCi) [1]. The time for determining the presence of RAI-refractoriness ranged from 6 to 60 months (median, 30 months). The control criterion, non-RAI-refractory disease, was an excellent response after RAI therapy with negative tumour mass imaging and a thyroglobulin (Tg) concentration < 0.2 ng/ml [16].

**Fig. 1:**
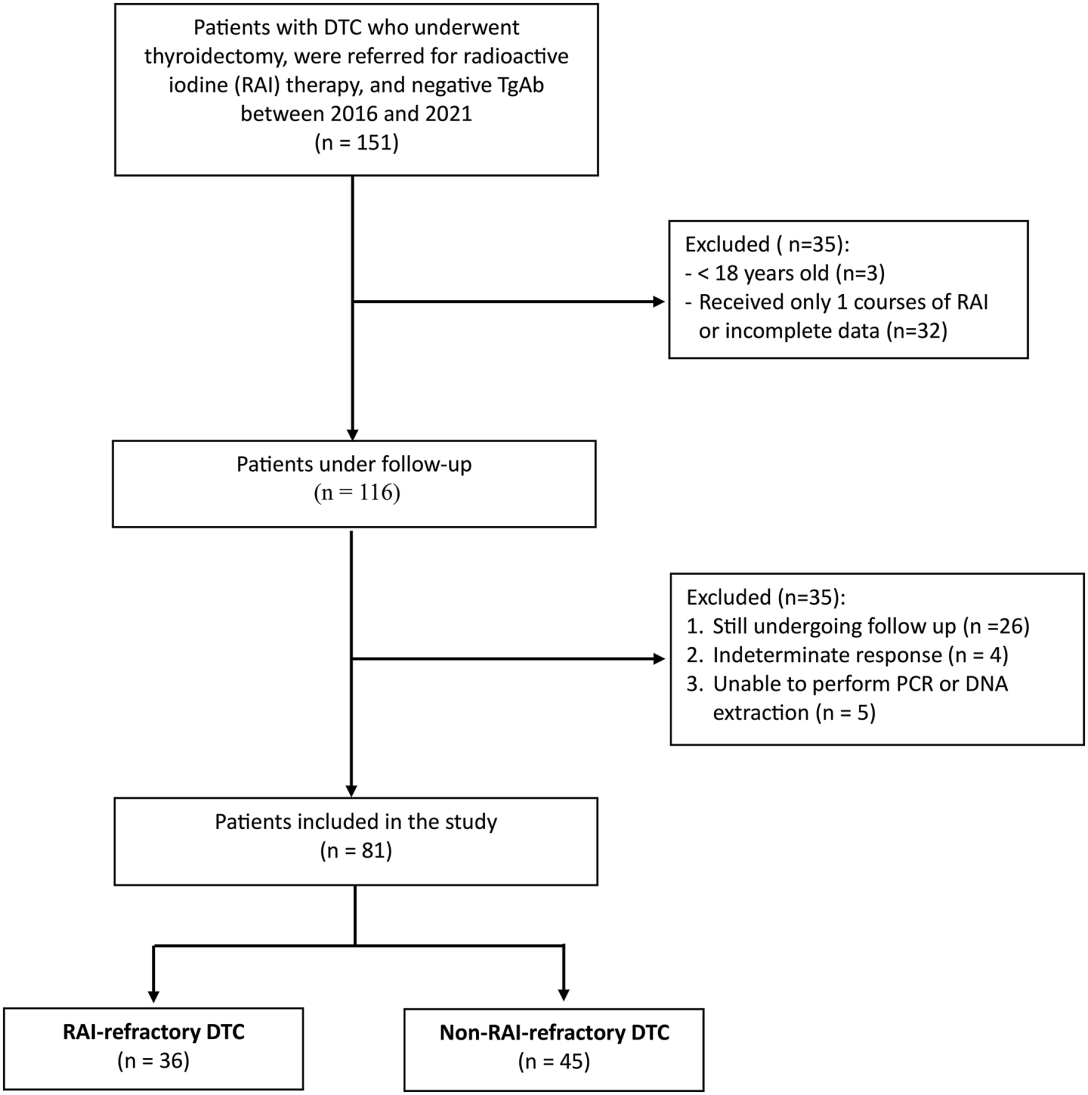
Flowchart illustrating patient selection process

To be eligible, participants had to meet specific criteria as follows: be 18 years of age or older, have undergone total or near-total thyroidectomy with or without neck dissection, have received at least 2 courses of RAI therapy, have analysable data, and have negative thyroglobulin antibody (TgAb) levels. A summary of patient selection is described in the flowchart (Fig. 1). Clinicopathological characteristics such as sex, age, histological type, aggressive subtype, TNM stage, lymphovascular invasion (LVI), and microscopic-extrathyroid extension (m-ETE) were obtained from medical records and reviews of haematoxylin and eosin (H&E) stained slides. The 5th edition of the World Health Organization (WHO) classification (2022) was used for histological types. The high-risk histologic group includes aggressive subtypes such as tall cell PTC, diffuse sclerosing PTC, hobnail PTC, columnar cell PTC, widely invasive FTC (including OCA), and PDTC [12, 17, 18]. The TNM staging system is based on the American Joint Committee on Cancer/Tumour-Node-Metastatic Staging System (AJCC/TNM) for Differentiated and Anaplastic Thyroid Cancer (Eighth Edition) 2017 [19]. Lymphovascular invasion (LVI) is defined as tumour cell invasion to blood vessels within the tumour capsule or beyond, with intravascular tumour cells attached to the wall and protruding into the lumen, covered by endothelium, or surrounded by fibrin in a fashion similar to that of an ordinary thrombus [17, 20]. Microscopic extrathyroidal extension (m-ETE) was defined as a tumour extending beyond the thyroid capsule into the surrounding peri-thyroidal soft tissues of fat and/or skeletal muscle without visual evidence of this invasion [21].

### Anexelekto (AXL) analysis

Immunohistochemical staining of the samples was performed using a labelled streptavidin-biotin immunoperoxidase complex to evaluate the expression of AXL. Samples were taken from formalin-fixed paraffin-embedded tissue, sectioned to 4-µm thicknesses, deparaffinized in xylene, and rehydrated using alcohol solution. Antigen retrieval was performed using a decloaking chamber at 96 °C for 20 min. Endogenous peroxidase blocking was performed with a 3% H2O2 solution for 10 min. Primary antibody incubation (1:1000, anti-AXL, Abcam, Catalogue No. ab219651, Cambridge, UK) was carried out for 1 h, followed by washing with phosphate-buffered saline (PBS) and secondary antibody incubation (Starr Trek Universal HRP Detection system, Biocare Medical, Concord, CA, USA) for 15 min. Finally, DAB substrate was added, and the sections were incubated for 5 min. Immunoexpression was evaluated by three pathologists using light microscopy, and the percentage of cells displaying membranous and cytoplasmic AXL immunostaining was determined. Tumour cells with less than or equal to 10% positivity were considered negative/low, while those with more than 10% positivity were considered high. At least five fields and more than 500 cells were analysed for each sample [6].

### *TERT* mutation analysis

Genomic DNA was extracted from 10-µm sections of formalin-fixed paraffin-embedded primary tumour samples using a commercial DNA extraction kit (Maxwell RSC FFPE Plus DNA Purification Kit, Promega, Catalogue No. AS1720, USA) according to the manufacturer’s protocol. The tumour samples were manually dissected under microscopic guidance by a pathologist, and any adjacent normal tissue surrounding the tumour was removed. The mutation threshold of the sample requires that at least 15–20% of cells harbor the gene mutations [22]. The regions harbouring two hotspot *TERT* promoter mutations, C228T and C250T, in the *TERT* gene were amplified by polymerase chain reaction (PCR) using the primers 5’-CCGTCCTGCCCCTTCACC-3’ (sense) and 5’-GGGCCGCGGAAAGGAAG-3’ (antisense). PCR amplification was performed with the following thermal cycling conditions: initial denaturation at 95 °C for 3 min, 35 cycles of denaturation at 95 °C for 30 s, annealing at 56 °C for 40 s, elongation at 72 °C for 50 s, and a final primer extension at 72 °C for 3 min. The PCR products were then confirmed for quality by agarose gel electrophoresis and subjected to Sanger sequencing analysis to determine the mutation status. Negative controls were included in every PCR to ensure contamination-free amplification.

### Statistical analysis

This study, with an observational case‒control design, involved data processing through SPSS version 24.0 for Windows. Statistical significance was determined using the Chi-square test, with alternative Kolmogorov‒Smirnov and Fisher’s exact tests as needed. Multivariate analysis of variables related to RAI-refractory status was conducted using binary logistic regression analysis.

## Results

### Patient characteristics

In this study, the overall prevalence of refractory RAI in DTC patients was 44.4% (36/81). The clinicopathological characteristics of the patients are shown in Table 1. The average patient age was 47 years, with 29.9% being males and 74.1% being females. The histological types of DTC included in the sample were high-grade follicular-derived carcinomas (involving differentiated high-grade thyroid carcinoma/DHGTC and PDTC), FTC, PTC, and invasive encapsulated follicular variant of papillary thyroid carcinoma (IEFVP). The most common histological type in this study was PTC (77.8%). Our research revealed different subtypes of PTC, including classic, encapsulated classic, infiltrative follicular, and clear cell subtypes, along with the IEFVP as a nonaggressive tumour with a low risk of RAI refractoriness. On the other hand, the tall cell PTC, columnar cell PTC, widely invasive FTC, DHGTC, and PDTC were categorized as high-risk histological groups due to their aggressive nature. Although the majority of the samples were classified as TNM stage I, the prevalence of LVI and m-ETE was high.

**Table 1:** Clinicopathological characteristics of patients

Variable	Total *N* (%)
Age (years)	
Mean ± SD	47.860 ± 14.436
≥ 40	60 (74.1)
< 40	21 (25.9)
Sex	
Male	21 (25.9)
Female	60 (74.1)
Histological type	
Follicular-derived carcinomas, high-grade	8 (9.9)
FTC	4 (4.9)
PTC	63 (77.8)
IEFVP	6 (7.4)
Aggressive subtype	
Aggressive	16 (19.8)
Nonaggressive	65 (80.2)
TNM Staging	
II-IV	20 (24.7)
I	61 (75.3)
LVI	
Yes	47 (58.1)
No	34 (41.9)
m-ETE	
Yes	43 (53.1)
No	38 (46.9)
AXL expression	
> 10% (high)	51 (63)
0–10% (negative/low)	30 (37)
*TERT* promoter	
Mutation	23 (28.4)
Wild-type	58 (71.6)

SD, standard deviation; FTC, follicular thyroid carcinoma; PTC, papillary thyroid carcinoma; IEFVP, invasive encapsulated follicular variant of papillary thyroid carcinoma; LVI, lymphovascular invasion; m-ETE, microscopic extrathyroid extension; AXL, anexelekto; TERT, telomerase reverse transcriptase

**Fig. 2:**
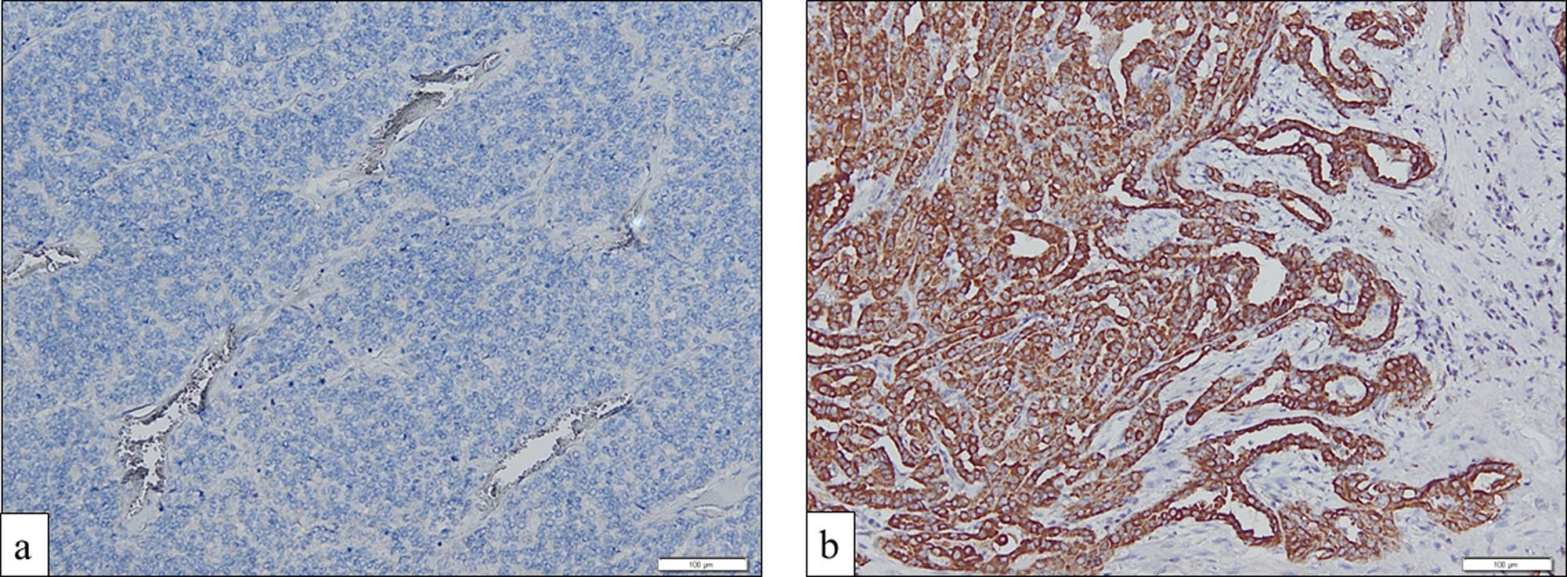
Immunohistochemical staining of anexelekto (AXL), (**a**) AXL Immunoexpression 0–10% (negative/low) (100x); (**b**) AXL immunoexpression > 10% (high) (100x)

Immunohistochemical staining revealed that AXL was primarily present in the membrane and cytoplasm of the tumour cells. After evaluation, AXL expression was high in 51 patients (63%) and negative/low in 30 patients (37%) (Fig. 2).

**Fig. 3:**
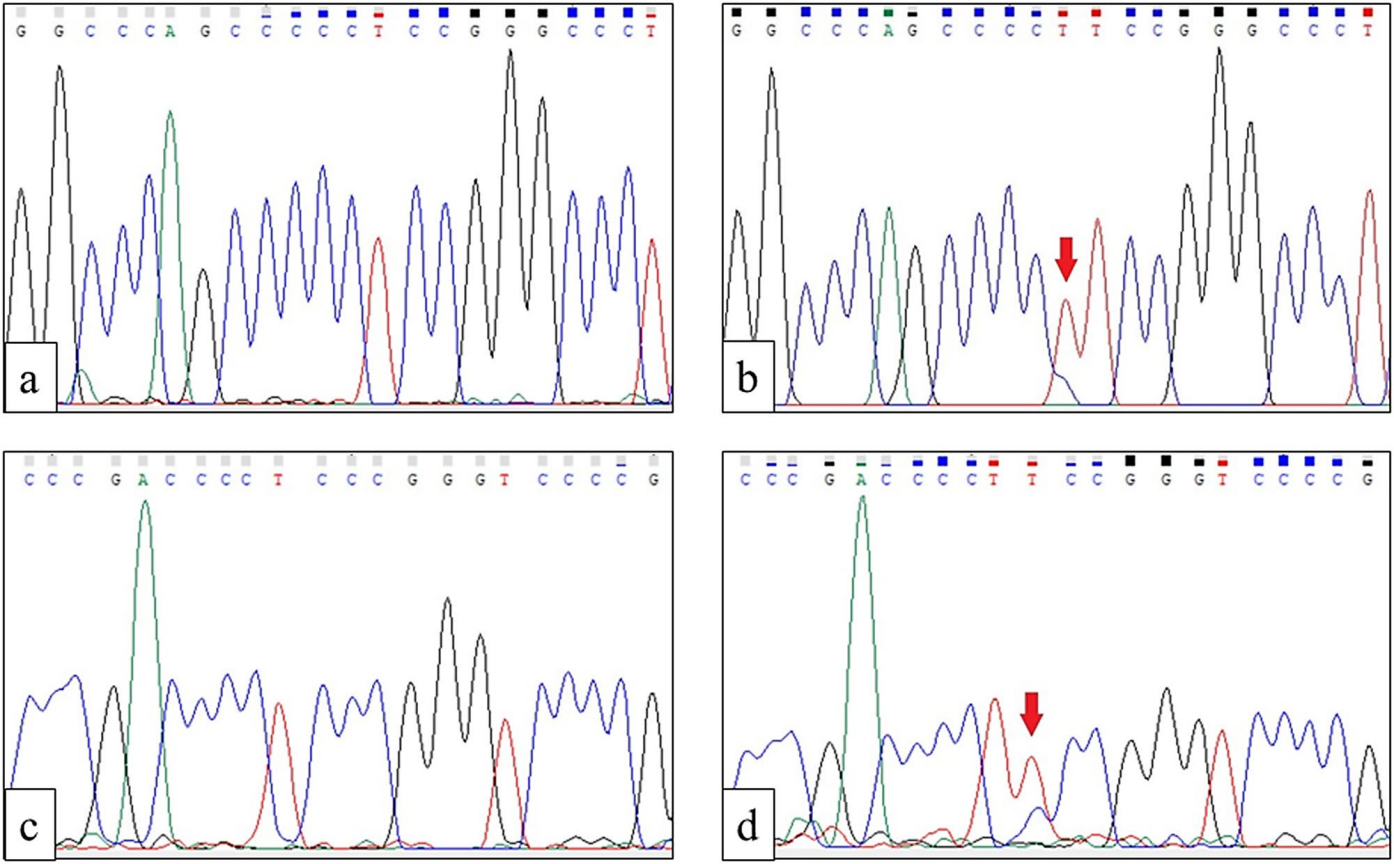
Sanger sequencing results for the two most common point mutations (hotspot regions) of the *TERT* promoter gene; (**a**) wild type; (**b**) mutation − 124 from transcription start site (TSS) C > T (C228T); (**c**) wild type; (**d**) mutation − 146 from transcription start site (TSS) C > T (C250T)

The *TERT* promoter sequencing results were analysed using Chromas version 2.6.6. We found that 23 samples (28.4%) had point mutations, including 17 with the C228T mutation and 6 with the C250T mutation (Fig. 3).

### Variables affecting RAI-refractory DTC

The clinicopathological characteristics, as well as AXL expression and *TERT* promoter mutation associated with RAI-refractory DTC, were analysed and are summarized in Table 2. Although the two groups had similar ages, sexes, and histological type distributions, univariate analysis revealed significant differences in clinicopathological characteristics, such as aggressive subtype, TNM stage, LVI, m-ETE, AXL expression, and *TERT* promoter mutation status (all p values < 0.01).

**Table 2:** Associations between clinicopathological characteristics and RAI-refractory DTC

Variable	RAI-refractory *N* (%)	Non-RAI-refractory *N* (%)	OR (95% CI)	*p* value
Age (years)				0.089
≥ 40	30 (83.3)	30 (66.7)	2.5 (0.89–7.31)	
< 40	6 (16.7)	15 (33.3)	1 (ref)	
Sex				0.061
Male	13 (36.1)	8 (17.8)	2.61 (0.94–7.27)	
Female	23 (63.9)	37 (82.2)	1 (ref)	
Histological type				0.066
Follicular-derived carcinomas, high-grade	7 (19.4)	1 (2.2)	14.00 (0.94–207.60)	
FTC	1 (2.8)	3 (6.7)	0.67 (0.039–11.29)	
PTC	26 (72.2)	37 (82.2)	1.41 (0.24–8.25)	
IEFVP	2 (5.6)	4 (8.9)	1 (ref)	
Aggressive subtype				< 0.001*
Aggressive	14 (38.9)	2 (4.4)	13.68 (2.85–65.64)	
Nonaggressive	22 (61.1)	43 (95.6)		
TNM staging				< 0.001*
II-IV	19 (52.8)	1 (2.2)	48.18 (6.10–396.52)	
I	17 (47.2)	44 (97.8)	1 (ref)	
LVI				< 0.001*
Yes	33 (91.7)	14 (31.1)	24.36 (6.38–93.01)	
No	3 (8.3)	31 (68.9)	1 (ref)	
m-ETE				< 0.001*
Yes	32 (88.9)	11 (24.4)	24.73 (7.14–85.62)	
No	4 (11.1)	34 (75.6)	1 (ref)	
AXL expression				
> 10% (high)	30 (83.3)	21 (46.7)	5.71 (1.99–16.40)	0.001*
0–10% (negative/low)	6 (16.7)	24 (53.3)	1 (ref)	
*TERT* promoter				
Mutation	21 (58.3)	2 (4.4)	30.10 (6.29–143.95)	< 0.001*
Wild-type	15 (41.7)	43 (95.6)	1 (ref)	

DTC, differentiated thyroid carcinoma; RAI, radioactive iodine; OR, odds ratio; CI, confidence interval; FTC, follicular thyroid carcinoma; PTC, papillary thyroid carcinoma; IEFVP, invasive encapsulated follicular variant of papillary thyroid carcinoma; LVI, lymphovascular invasion; m-ETE, microscopic extrathyroid extension; AXL, anexelekto; TERT, telomerase reverse transcriptase; ref, reference

*Statistically significant difference

The multivariate analysis results are presented in Table 3. We used a binary logistic regression model to determine the adjusted odds ratio (OR) associated with RAI-refractory DTC, considering six factors. Among these six variables, TNM stage, LVI, and m-ETE did not significantly differ. However, only the aggressive subtype (adjusted OR: 74.75, 95% confidence interval [CI]: 1.31-4266.20, *p* = 0.037), high AXL expression (adjusted OR: 44.98, 95% CI: 1.41-1439.03, *p* = 0.031), and *TERT* promoter mutation (adjusted OR: 10.95, 95% CI: 1.06-112.92, *p* = 0.044) were independently associated with RAI-refractory status.

**Table 3:** Multivariate analysis of variables related to RAI-refractory DTC based on binary logistic regression analysis

Variable	Adjusted OR (95% CI)	*p* value
Aggressive subtype		
Aggressive	74.75 (1.31–4266.20)	0.037*
Nonaggressive	1 (ref)	
TNM staging		
II-IV	8.05 (0.60–108.09)	0.116
I	1 (ref)	
LVI		
Yes	2.69 (0.43–16.96)	0.291
No	1 (ref)	
m-ETE		
Yes	5.44 (0.92–32.07)	0.061
No	1 (ref)	
AXL expression		
> 10% (high)	44.98 (1.41–1439.03)	0.031*
0–10% (negative/low)	1 (ref)	
*TERT* promoter		
Mutation	10.95 (1.06–112.92)	0.044*
Wild-type	1 (ref)	

DTC, differentiated thyroid carcinoma; RAI, radioactive iodine; OR, odds ratio; CI, confidence interval; LVI, lymphovascular invasion; m-ETE, microscopic extrathyroid extension; AXL, anexelekto; TERT, telomerase reverse transcriptase; ref, reference

*Statistically significant difference

## Discussion

In this study, univariate analysis revealed that 6 variables significantly increased the risk of RAI-refractory DTC: histopathological aggressive tumour subtype, TNM stage II and above, LVI presence, m-ETE, high AXL expression, and *TERT* promoter mutation (all p values < 0.05). However, multivariate analysis showed only the aggressive tumour subtype, high AXL expression, and *TERT* promoter mutation were independently associated with RAI-refractory status.

TNM staging for patients under 55 years old, based on the AJCC 8th edition, is divided into two groups: stage I (without metastases) and stage II (with metastases). We grouped the stages into two groups: stage I and stage II-IV. Patients with more advanced disease (stage II-IV) have a potential risk of RAI-refractory disease as demonstrated in studies by Meng et al. [23] and Li et al. [24]. This is because as the stage increases, the cells become dedifferentiated, leading to more aggressive growth, metastases, and decreased uptake of iodide, making them unresponsive to RAI therapy [25].

Extrathyroidal extension (ETE) refers to the spreading of a thyroid tumour beyond the thyroid capsule into adjacent soft tissue [21]. ETE can be divided into two types: minimal (microscopic), which involves the sternothyroid muscle or peri-thyroidal soft tissues and is identified by light microscopic examination, and advanced (extensive), which is defined as a direct extension of the tumour into subcutaneous soft tissues, larynx, trachea, oesophagus, or recurrent laryngeal nerve [26]. The current guidelines of the American Thyroid Association (ATA) and European Thyroid Association (ETA) state that m-ETE is a moderate risk factor for disease recurrence and/or persistence [26, 27]. In contrast, the 8th edition of the AJCC TNM defines T3b as a widespread extrathyroidal extension to the muscle, but it does not include a minimal extension through the thyroid capsule (m-ETE) in the staging. However, Parvathareddy et al. [21] reported that m-ETE is significantly associated with adverse clinicopathological characteristics, including age ≥ 55 years, male sex, tall cell PTC subtype, bilateral tumours, multifocality, lymphovascular invasion, regional lymph node metastases, distant metastases, tumour recurrence, and poor RAI therapy response.

Although the odds ratio for TNM staging in this study (OR: 8.05, 95% CI: 0.60–108.09, *p* = 0.116) indicates a potential association, with patients exhibiting TNM staging II-IV being eight times more likely to experience RAI refractoriness compared to those with stage I, the wide confidence interval and non-significant p-value indicate considerable uncertainty in this association. Similarly, m-ETE showed a potential link with RAI refractoriness (OR: 5.44, 95% CI: 0.92–32.07, *p* = 0.061), but the wide confidence interval and borderline statistical significance limit its predictive reliability. These findings highlight potential clinical trends consistent with the biological behavior of aggressive thyroid cancers but underscore the need for further research with larger sample sizes to clarify these associations and confirm their predictive value.

Certain histological subtypes of DTC, such as tall cell and diffuse sclerosing PTC, in a study by Pyo et al. [28], have lower NIS expression and are unable to concentrate RAI, making them RAI refractory. Additionally, hobnail PTC is linked to *TERT* promoter mutation, causing it to become more aggressive and often resistant to RAI treatment. Approximately 50% of PDTCs and DHGTCs, which have an intermediate prognostic risk according to the WHO 2022 classification, are positive for FDG-PET and are less concentrated in the RAI [29]. This change towards dedifferentiation reduces the ability of tumour cells to take up RAI, resulting in a RAI-refractory status. In epigenetic terms, TSHR hypermethylation, the NIS gene promoter, and histone acetylation can reduce the effectiveness of RAI therapy [24]. Our analysis revealed that a more aggressive subtype has a significant impact on the occurrence of RAI-refractory DTC.

This study revealed that compared with low/negative AXL expression, high AXL expression in tumour cells is positively correlated with RAI-refractory development. AXL is an essential protein characterized by extracellular, transmembrane, and intracellular domains. Encoded by the *AXL* gene, this protein is a member of the TAM family of receptor tyrosine kinases [30]. Collina et al. [6] reported that high AXL expression levels were significantly associated with RAI-refractory status, disease persistence/recurrence, and reduced disease-free survival (DFS) in human PTC cohort samples. Wei et al. [7] reported that inhibiting the PROS1-AXL-mediated TAM signalling pathway with an AXL inhibitor could suppress the proliferation and migration of human PTC cells. RAI reversal occurs due to the loss of the function of NIS, an iodide symporter, and this loss of function is often caused by oncogenic signalling pathways, including the activation of aberrant receptor tyrosine kinases; in this study, AXL was shown to stimulate the RTK/BRAF/MEK/ERK or PI3K/AKT1 transduction pathways. These pathways can suppress thyroid-specific transcription factors (TTFs), such as PAX8, a positive regulator of NIS transcription [6]. Activation of certain pathways, such as the PI3K/AKT/mTOR pathway or loss of PTEN, can inhibit NIS glycosylation and localization to cell membranes [31]. Cabozantinib, a multikinase inhibitor that targets VEGFR, MET, FLT3, c-Kit, and AXL, has been shown to be effective in treating RAI-refractory DTC patients who previously received lenvatinib or sorafenib and disease that progresses during or after treatment with two or more VEGFR tyrosine kinase inhibitors. Currently, cabozantinib is undergoing phase III clinical trials as a monotherapy for RAI-refractory DTC [3, 32].

Several research has focused on molecular mutations, notably *TERT* promoter mutation and *BRAF*^*V600E*^, in the context of radioiodine-refractory differentiated thyroid carcinoma (DTC), offering critical insights into the genetic mechanisms driving treatment resistance. A synthesis of findings from a range of studies confirms that the *TERT* promoter mutation is a robust predictor of resistance to treatment, as consistently observed across multiple investigations. This mutation is strongly associated with more aggressive tumor phenotypes and less favorable clinical outcomes. Its presence is reliably linked to reduced iodine uptake and diminished response to radioiodine therapy, underscoring its pivotal role in forecasting treatment refractoriness [13, 33–36]. Conversely, the predictive capacity of *BRAF*^*V600E*^ mutations appears to be less consistent, potentially due to factors such as tumor heterogeneity and the presence of other concurrent mutations. Data aggregated from studies by Tan et al. [37], Pyo et al. [35], and Crezee et al. [36] indicate that the *BRAF*^*V600E*^ mutation alone does not consistently correlate with radioiodine-refractory thyroid carcinoma.

Patients with *TERT* promoter mutation tend to develop RAI-refractory disease. Similar results were reported by Yang et al. [13], who reported that *TERT* promoter mutation was closely correlated with RAI-refractory disease, with a high specificity of 100% and a positive predictive value of 100%, and showed a significant effect on poor clinical outcome. In the study by Crezee et al. [36], the proportion of tumours harbouring *TERT* promoter mutation was significantly greater in the RAI-refractory group than in the RAI-avid group (50% vs. 8.6%). The *TERT* oncogene plays several roles in the development and progression of cancer cells. TERT/telomerase activation is required for cell transformation and unlimited proliferation by stabilizing telomere length [38]. In addition, TERT activity outside the nucleus contributes to the main regulatory mechanism within cells, including survival, gene expression, signal transduction pathways, and mitochondrial metabolism. In the regulation of mitochondrial metabolism, TERT can reduce oxidative stress, reduce the rate of DNA damage, and reduce apoptosis, resulting in treatment resistance [15]. Currently, this molecular marker is not recommended for postoperative prognostic factors or for the management of DTC, but this marker can provide significant value in treating RAI-refractory disease compared with conventional treatment protocols [18].

The role of AXL in cell proliferation and survival is established through its activation of the MAPK/ERK, PI3K, and JNK pathways [39]. Moreover, AXL’s heterodimeric interactions especially with EGFR (HER2) can strategically shift downstream signaling from PI3K to MAPK [40]. The activated MAPK/ERK pathway plays a crucial role in enhancing the interaction between Sp1 and GABPA by phosphorylating Sp1 and dissociating it from HDAC1, thereby stabilizing GABPA’s binding to the *TERT* promoter mutant [41]. This interplay results in synergistic effects between the AXL receptor tyrosine kinase and the *TERT* promoter mutant on cell proliferation and survival.

*TERT* promoter mutation is typically associated with the later stages of carcinogenesis, while *BRAF* and *RAS* mutations are encountered earlier in the process [38]. *BRAF*^*V600E*^ oncogene reduces NIS mRNA transcription levels and epigenetically regulates NIS by inducing histone deacetylation in the NIS promoter, effectively inhibiting its transcription [24]. *RAS* mutations cause intrinsic defects that disrupt GTP hydrolysis and resistance to GTPase-activating proteins (GAPs), enabling RAS mutants to activate both the MAPK and PI3K pathways simultaneously [42]. Furthermore, Tan et al. [37] reported that patients with *BRAF*^*V600E*^ and *TERT* promoter mutations progressed more rapidly to RAI-refractory DTC than those with *BRAF*^*V600E*^ alone. Detecting TERT promoter mutations may aid in early diagnosis, prognosis, and treatment decisions for RAI-refractory DTC.

This retrospective study has several limitations. The primary limitation is the relatively small sample size and short follow-up duration. Extended follow-up data for long-term survival and recurrence rates are lacking. Additionally, data on other significant molecular mutations, such as BRAF and RAS, are unavailable, limiting broader comparisons and in-depth analysis of molecular pathways. A larger cohort study with a longer follow-up period is necessary. Further research is required to explore the clinical implications of these two molecular markers, particularly regarding their association with poorer therapy responses and outcomes.

## Conclusions

Our predictive model suggested that high AXL expression, *TERT* promoter mutation, and aggressive histopathological subtypes are associated with RAI-refractory disease and may be of value in adjusting treatment decisions. Additionally, AXL has the potential to be a targeted therapy for RAI-refractory disease.

## Data Availability

The datasets used and/or analysed during the current study are available from the corresponding author on reasonable request.

## Abbreviations

AJCC/ TNM: American Joint Committee on Cancer/Tumour-Node-Metastatic
ATA: American Thyroid Association
AXL: Anexelekto
BRAF: B-type Rapidly Accelerated Fibrosarcoma
DHGTC: Differentiated high grade thyroid carcinoma
DNA: Deoxyribonucleic Acid
DTC: Differentiated Thyroid Carcinoma
ETS: E-twenty‐six
FTC: Follicular Thyroid Carcinoma
H&E: Haematoxylin and Eosin
IEFVP: Invasive encapsulated follicular variant of papillary thyroid carcinoma
LVI: Lymphovascular invasion
MAF: Mutant allele fraction
mCi: Millicurie
m-ETE: Microscopic-extrathyroid Extension
OCA: Oncocytic Carcinoma
OR: Odds Ratio
PBS: Phosphate-buffered Saline
PCR: Polymerase Chain Reaction
PDTC: Poorly Differentiated Thyroid Carcinoma
PTC: Papillary Thyroid Carcinoma
RAI: Radioactive Iodine
RECIST: Response Evaluation Criteria in Solid Tumours
TERT: Telomerase Reverse Transcriptase
Tg: Thyroglobulin
TgAb: Thyroglobulin Antibody
TNM: Tumour Nodes Metastases
TSH: Thyroid-stimulating Hormone
TSS: Transcription Start Site
WHO: World Health Organization
